# Effect of turmeric on colon histology, body weight, ulcer, IL-23, MPO and glutathione in acetic-acid-induced inflammatory bowel disease in rats

**DOI:** 10.1186/s12906-016-1057-5

**Published:** 2016-02-23

**Authors:** Salim M. A. Bastaki, Mohammed Majed Al Ahmed, Ahmed Al Zaabi, Naheed Amir, Ernest Adeghate

**Affiliations:** Department of Pharmacology, College of Medicine and Health Sciences, United Arab Emirates University, P.O. Box 17666 Al Ain, UAE; Department of Anatomy, College of Medicine and Health Sciences, United Arab Emirates University, P.O. Box 17666 Al Ain, UAE

**Keywords:** Inflammatory bowel disease, Rat, Histopathology, *Curcuma longa*, Ulcer

## Abstract

**Background:**

This study investigates the protective effects of turmeric (*Curcuma longa*, CL) on acetic acid-induced colitis in rats.

**Method:**

Inflammatory bowel disease (IBD) was induced in male Wistar rats by intra-rectal administration of 1 ml of 4 % acetic acid at 8 cm proximal to the anus for 30 s. Curcuma longa (CL) powder, (1, 10, or 100 mg/kg/day) was administered for either 3 days before or after IBD for 7 days. The body weight, macroscopic and microscopic analysis of the colon of CL-treated IBD rats and that of control rats (no IBD, no CL) were performed on 0 day, 2, 4 and 7th day. Myeloperoxidase (MPO), IL-23 and glutathione levels in control, untreated and treated rats were measured by ELISA.

**Results:**

CL significantly (*P <* 0.05) improved IBD-induced reduction in mean body weight and mean macroscopic ulcer score. Administration of CL also significantly (*P <* 0.01) reduced the mean microscopic ulcer score when compared to untreated IBD control. Intake of CL by rats resulted in a significant (*P <* 0.05) increase in the mean serum glutathione level compared to untreated control. CL reduced both MPO and IL-23 levels in the colonic mucosa of the rat.

**Conclusion:**

CL improved body weight gain, mean macroscopic and microscopic ulcer scores in the colon of rats suffering from acetic acid-induced IBD. CL reduced both MPO and IL-23 in the mucosa of the colon. The increase in the mean serum glutathione level may help in the reduction of oxidative stress associated with IBD.

## Background

Inflammatory bowel disease (IBD) is characterized by recurrent ulceration of the bowel and is of unknown etiology [1]. The pathogenesis likely involves genetic, environmental, and immunologic factors [2, 3]. Genetic deletion or antibody-mediated neutralization of interleukin 12 (IL-12) led to amelioration of intestinal inflammation in a number of different models [4, 5]. The discovery in 2000 of a related cytokine interleukin (IL) 23, has, however, led to the re-evaluation of the functional role of IL-12 in inflammation. In fact, it was shown that IL-23 and not IL-12 is the key molecule in the development of a variety of inflammatory conditions including intestinal inflammation [6]. The demonstration of the association of genetics in the IL-23 pathway in multiple chronic inflammatory disorders, including inflammatory bowel disease (IBD), has coincided with significant advances in the understanding of its key role in host defense and organ-specific autoimmunity. Evidence for the importance of IL-23 pathway in IBD has come from mouse models of IBD, in which IL-23 deficiency or blockade protects not only the mice from the disease [7, 8], but human IBD as well [9–11].

Treating IBD and limiting drug toxicity is a continuous challenge. The drugs of choice for the treatment of IBD are 5-aminosalicylic acid and sulfasalazine. Others include corticosteroids, azathioprine, mercaptopurines and cyclosporine, which are used for more severe forms of the disease [12]. There is a pressing need for developing new and effective therapeutic approaches with limited toxicity. The use of medicinal plants or their active components is becoming an increasingly attractive approach for the treatment of various inflammatory disorders among patients unresponsive to steroids or unwilling to take standard medications. Food derivatives are increasingly being used as therapy for IBD because of limited toxicity. During the last ten years, a large number of dietary components have been evaluated as potential chemo-preventive agents [13]. Turmeric, the powdered rhizome of the medicinal plant, *Curcuma longa* Linn, of the Zingiberaceae family is widely used as a food flavouring and colouring agent in the Asian cuisine. It grows naturally throughout the Indian subcontinent and in tropical climates. India produces most of the world’s turmeric [14, 15]. Its yellow colour is due to curcumin (diferuloylmethane), a polyphenolic pigment [16]. Curcumin has been used since ancient times for its medicinal property in India and Southeast Asia and is known to possess a variety of pharmacological effects including anti-inflammatory activities. Studies by Chuang et al. showed that curcumin, the active ingredient of turmeric strongly inhibited drug-induced inflammation in the liver [17]. It was also shown that curcumin was able to inhibit NF-Kappa B, the molecule responsible for the proliferation of inflammatory cytokines in colitis [18]. In this particular study, curcumin made up 2.0 % of the daily feed of the Wistar rats for a period of two weeks [18]. Mucosal damage was also reported to be improved in colitis induced by trinitrobenzene sulphonic acid [19]. Extract of the turmeric has also been reported to have myorelaxant effect in addition to anti-inflammatory action on animal models of colitis [20]. Since turmeric is widely used as a flavouring agent in food and because of its medicinal properties, it was thought worthwhile to investigate the protective effects of turmeric (*Curcuma longa*, CL) on acetic acid-induced colitis in a rat model of IBD with special emphasis on colon ulcers and lymphatic infiltration.

## Methods

### Animals

Male Wistar rats weighing between 225–240 g were used for the experiments. Rats were housed under normal laboratory conditions set at 21–24 °C and 40–60 % relative humidity, under 12 h light/dark cycle with free access to standard rodent food and water. For each set of experiments, the animals were divided into three groups, control (no IBD, no CL); untreated (IBD, no CL); treated (IBD, CL). The experimental protocols of this study were approved by the Institutional Animals Ethical Committee of the United Arab Emirates University (Ethical Approval # A6-15). All experiments were performed according to ethical guidelines set by the United Arab Emirates University.

### Induction of colitis

After an overnight fasting, the rats were lightly anaesthetized with ether and IBD was induced by intrarectal administration of 1 ml of 4 % acetic acid at 8 cm proximal to the anus for 30 s. To flush the colon, 1 ml of phosphate buffered saline (PBS) was similarly administered.

### Pharmacological treatment

CL powder (1, 10, or 100 mg/kg/day), purchased from Mehran Spice & Food Industries, Plot # 14 & 15 Sector 24, Korangi Industrial Area, Karachi, Pakistan, was administered orally for 3 days before or 30 min after the induction of IBD. Control animals received 1 ml/kg saline using the same technique. The animals were weighed at 0 day, 2, 4 and 7 days after IBD in both control (no IBD nor CL treatment) and in CL-treated IBD rats.

### Macroscopic evaluation of colonic damage

To examine the extent of colonic inflammation by macroscopic and microscopic analysis, histological samples were collected at selected time points (0 day, after 3 days of treatment and after 2, 4 and 7 days of IBD with or without CL treatment). Briefly, immediately after the 7th day of IBD, the rats were euthanized by cervical dislocation and the colon excised 8 cm above the anal margin, opened longitudinally and washed with saline. Macroscopic damage was assessed by the scoring system of Wallace and Keenan [21], which takes into account the area of inflammation and the presence or absence of ulcers. Briefly, the criteria for assessing macroscopic damage was based on a semi quantitative scoring system where features are graded as follows: 0, no ulcer, no inflammation; 1, no ulcer, local hyperaemia; 2, ulceration without hyperaemia; 3, ulceration and inflammation at one site only; 4, two or more sites of ulceration and inflammation; 5, ulceration extending more than 2 cm.

### Histology

After macroscopic observation, samples of the colon were subsequently excised for microscopic observation. Microscopic analysis was based on the scoring system of Neurath et al., [5]. The colon was fixed in 10 % formalin in phosphate buffered saline (PBS) for 1 week after which it was washed under running tap water for 2 h. The samples were then dehydrated in graded ethanol and then embedded in paraffin wax. Thereafter, the sections were deparaffinized with xylene, stained with haematoxylin-eosin and were viewed microscopically. Lymphatic infiltration was scored as follows: none; confined to the mucosa; mucosa and submucosa; traverses the whole length of the colonic wall.

### Determination of myeloperoxidase

Myeloperoxidase (MPO) was measured by sandwich ELISA according to manufacturer’s protocols. Briefly, 100 μl of the sample and standards were added to the 96 well microtiter plate, coated with antibodies recognizing rat MPO, for 1 h at room temperature. After washing, 100 μl /well biotinylated trace antibody was added for 1 h at room temperature. After washing, Streptavidin-peroxidase conjugate was added to bind with biotinylated trace antibody for 1 h at room temperature. The TMB-ELISA substrate was added for 30 min at room temperature, after washing of the plate. The enzyme reaction was stopped by oxalic acid. The absorbance was read at 450 nm, after adding stop solution, with a microplate reader (Tecan Group Ltd., Männedorf, Switzerland). MPO levels were expressed as ng per milligram of protein.

### Determination of IL −23

Enzyme immunoassay of IL-23, in distal colon protein samples was performed by using commercial Six C USA sandwich ELISA kit. Briefly, sample and standards were added to the coated 96 well micro titer plate for 30 min at 37 °C. After washing, HRP was added for at 37 °C. The TMB-ELISA substrate was added, after washing. The absorbance was read at 450 nm, after adding a stop solution, with a microplate reader (Tecan Group Ltd., Männedorf, Switzerland). IL −23 levels were expressed as pg per milligram of protein.

### Determination of glutathione

Enzyme immunoassay of glutathione (GSH) in serum was performed by using Six C USA Sandwich ELISA kit. Briefly, sample and standards were added to the coated 96 well micro titer plate for 30 min at 37 °C. The procedure was performed according to the guidelines given by the vendor (Tecan Group Ltd., Männedorf, Switzerland). Serum GSH levels were expressed as μM per litre of blood.

### Protein extraction for ELISA

Samples of 8 cm portion of distal colon were cut longitudinally to open, washed with ice-cold PBS, and the mucosa was rapidly scraped from the underlying tissue layers on ice. The mucosa was weighed, minced by forceps, and homogenized with 10 volumes of in ice-cold high KCl lysis buffer 10 mM Tris–HCl, pH 8.0, 140 mM NaCl, 300 mM KCl, 1 mM EDTA, 0.5 % Triton X-100 and 0.5 % sodium deoxycholate with complete protease inhibitor cocktail with a polytron homogenizer (IKA laboratory, Germany). The resulting homogenates, after 30 min incubation on ice, were centrifuged at 15000 rpm for 30 min at 4 °C. The resulting supernatant was stored at −80 °C until the ELISA was performed. Protein concentration was determined in each sample by the BCA method based commercial kit.

### Chemicals and kits

Acetic acid was purchased from BDH Prolabo (10 Village Crescent, Linbro Business Park, Linbro Park, 2065, Johannesburg, South Africa). Sodium deoxycholate and all other chemicals, if not specified were purchased from Sigma-Aldrich Chemical Company (Sigma Chemical Co., St. Louis, MO, USA).

BCA Protein Assay kit and complete protease inhibitor cocktail were purchased from Thermo Fisher Scientific Inc. (81 Wyman Street, Waltham, MA USA 02451). Rat MPO sandwich ELISA kit was purchased from Hycult Biotech (Frontstraat 2a, 5405 PB Uden, The Netherlands). Rat IL-23 ELISA kit was purchased from 6C USA Inc. (Ausmausco Pharma, Co., Ltd. China). Glutathione ELISA kit was procured from Tecan Group Ltd., Männedorf, Switzerland.

### Statistical analysis

SPSS software 20 was used for the statistical analysis. Data were expressed as means ± S.E.M. Mann–Whitney *U* test was used for non-parametric. P values ≤ 0.05 were regarded as significant.

## Results

### Effect of curcuma longa (CL) on body weight in acetic acid-induced inflammatory bowel disease (IBD)

**Fig. 1:**
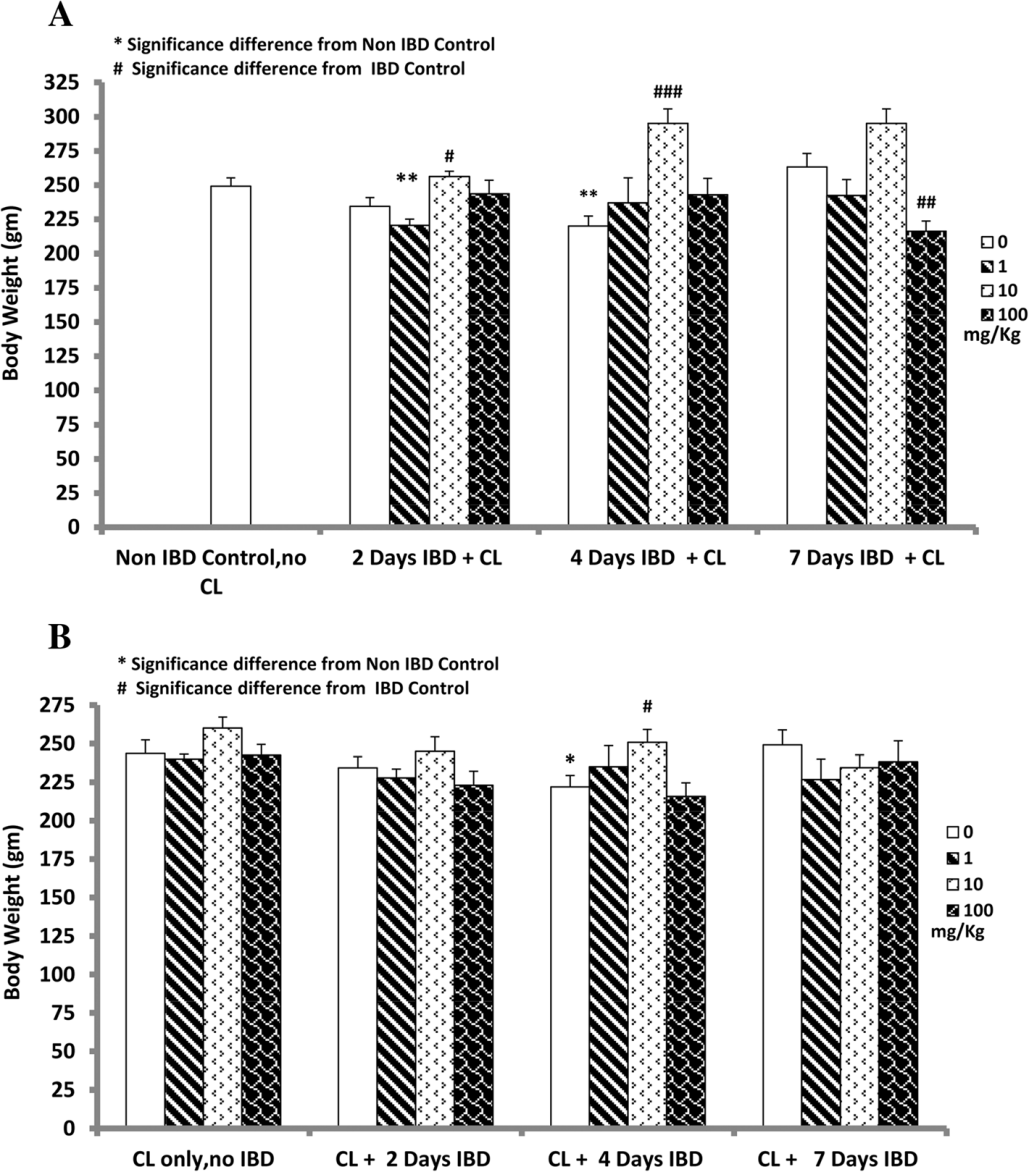
Figure 1 shows the mean body weight (MBW) of rats treated with C*urcuma Longa* (CL) before (**b**) and after (**a**) IBD. Note that CL at a dose of 10 mg/kg significantly (*P <* 0.05) increased the MBW on days 2 and 4 after the onset of IBD, (*n =* 5–6). *Significance difference between CL-treated and non IBD control; # Significance difference between CL-treated and IBD control

The mean body weight (MBW) in controlled rats (before IBD) was 249 ± 6.0 g (*n =* 11). After 4 days of IBD, the MBW of the untreated rats decreased significantly (*P <* 0.05) to 220 ± 7.0 g (*n =* 10) but recovered by day 7 (263 ± 10.0, *n =* 11). CL at a dose of 10 mg/kg significantly (*P <* 0.05, *P <* 0.01) increased the MBW to 256 ± 4.0 g on day 2 after the onset of IBD, (*n =* 5) and to 295 ± 11.0 g on day 4 of IBD (*n =* 6) when compared to untreated IBD rats (Fig. 1).

The body weight of rats pre-treated with CL by gastric gavage at doses of 1, 10 and 100 mg/kg per day for 3 days before the induction of IBD is presented in Fig. 1b. The MBW of control rats was not significantly affected by IBD on days 2 and 7. However, a significant reduction in MBW was seen on day 4 of IBD (222 ± 7.0 g) compared to control (243 ± 5.0 g). In contrast, the MBW of CL-pre-treated rats with 10 mg/kg, increased significantly on day 4 after the onset of IBD from 222 ± 7.0 g (untreated rats) to 251 ± 8.0 g (*n =* 6)(Fig. 1a1) . CL at doses of 1 and 100 mg/kg/day had no significant effect on MBW at days 2 and 7 of IBD compared to untreated IBD rats.

### Effect of curcuma longa (CL) on mean macroscopic ulcer and score (MaUS) in acetic acid-induced inflammatory bowel disease (IBD)

**Fig. 2:**
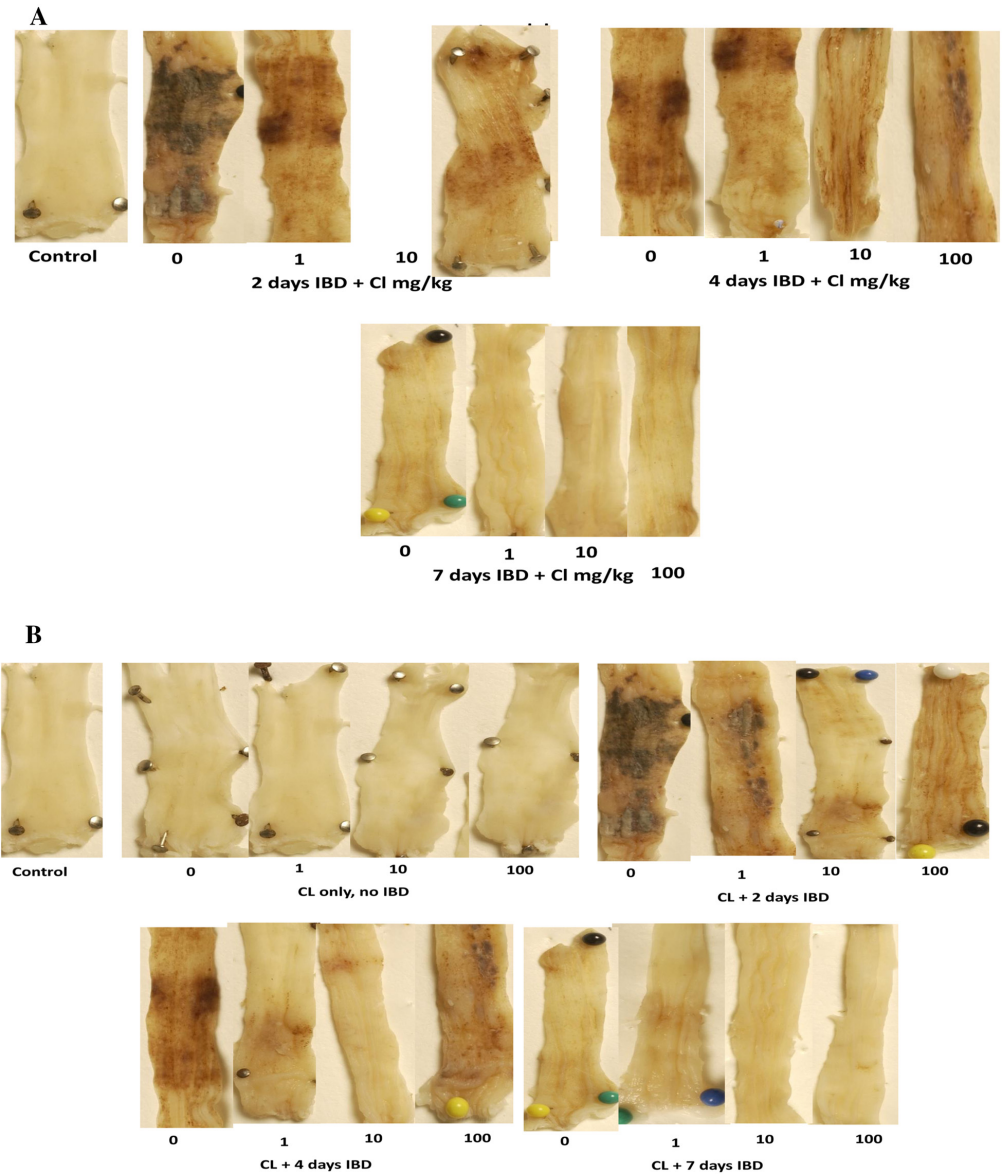
Figure 2 shows the effect of different doses of C*urcuma Longa* (CL) on colonic mucosa before (**b**) or after (**a**) the onset of acetic acid-induced IBD. The hyperaemia and ulcers caused by IBD in the colon of these rats were less severe compared to those that were treated with CL after the onset of IBD. 10 mg/kg of CL appears to have the most beneficial effect (*n =* 6)

**Fig. 3:**
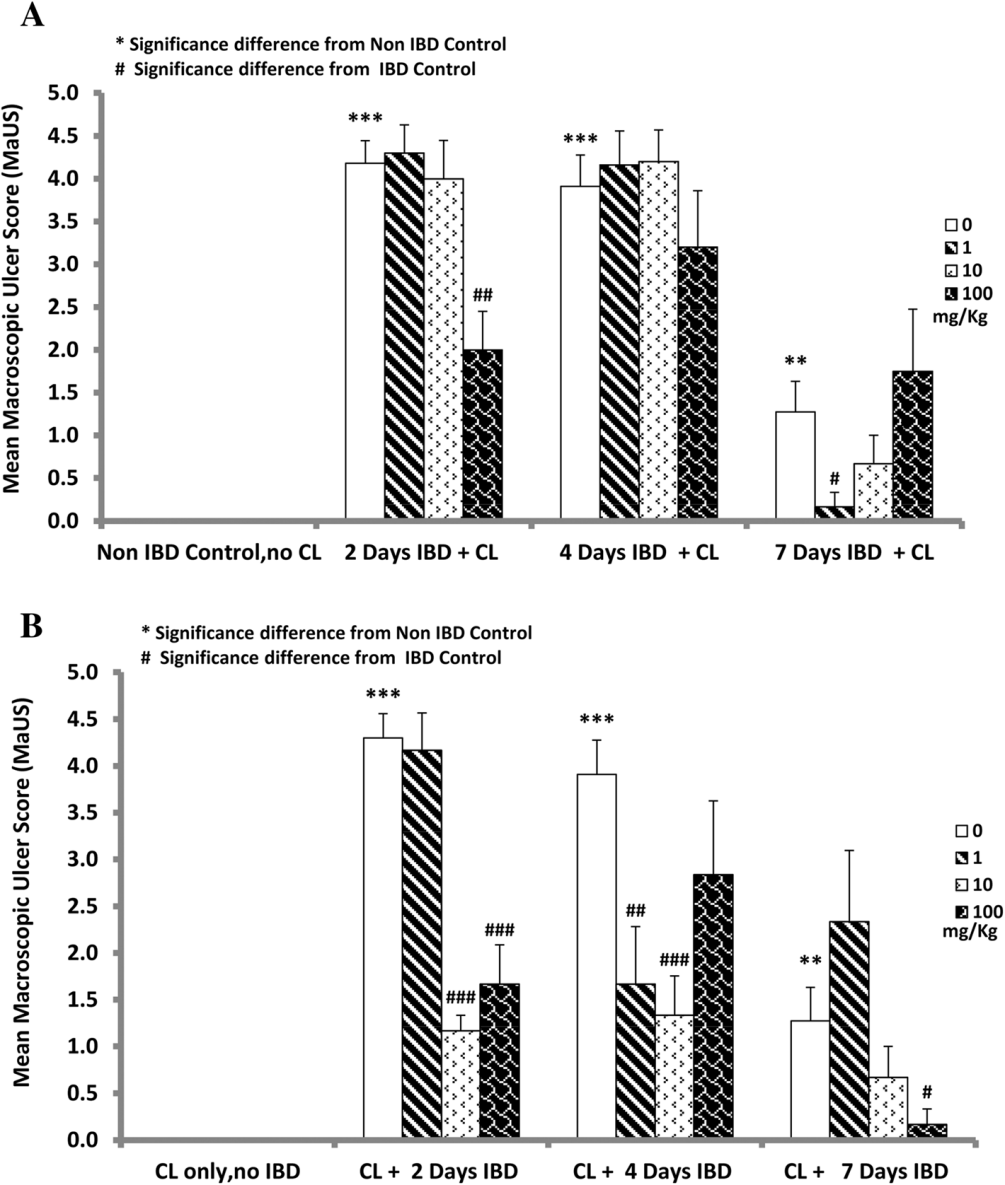
Figures 3 shows the effect of orally administered C*urcuma Longa* (CL) on the macroscopic ulcer score (MaUS) in the colon of rat before (**b**) and after (**a**) the induction of IBD. Note that CL at a dose of 1 and 10 mg/kg, caused a significant (*P <* 0.05) decrease in MaUS after day 7, when compared to control. In the pretreated group, CL at a doses of 1, 10 and 100 mg/kg, resulted in a significant (*P <* 0.001) reduction in MaUS *albeit* at different time points. *Significance difference between CL-treated and non IBD control; # Significance difference between CL-treated and IBD control *n =* 6

The effect of CL on MaUS in rats when given orally immediately after the induction of IBD is depicted in Fig. 2. The mucosa of the colon of untreated rats is hyperemic and ulcerative. Scoring (Fig. 3) of the lesions of the hyperemia and ulcer of the colon of acetic acid-induced IBD rats showed significant (*P <* 0.001) increase in MaUS from 0 (control) to 4.2 ± 0.26, 3.9 ± 0.4, and 1.3 ± 0.4 after 2, 4 and days 7, respectively. CL at a dose of 1 mg/kg, caused a significant decrease in MaUS after day 7 (0.2 ± 0.02) when compared to untreated groups, 4.2 ± 0.26 (IBD, no CL, *n =* 6). CL, when administered at a dose of 100 mg/kg also caused a significant (*P <* 0.01) reduction in MaUS after 2 days of IBD from 4.2 ± 0.26 (untreated) to 2.0 ± 0.45. However, CL administered at a dose of 100 mg/kg had no significant effect on MaUS after 4 and 7 days of IBD when compared to control (IBD, no CL).

The effect of CL on hyperemia and ulcer in the mucosa of the colon of control, untreated, and treated rats is presented in Fig. 2. Note that 10 mg of CL is the most effective dose in the prevention of hyperemia and ulceration. IBD caused a significant increase in MaUS from 0 (untreated) to 4.3 ± 0.3 (*n =* 6), 3.9 ± 0.4, (*n =* 6) and 1.3 ± 0.4 at days 2, 4 and 7, post IBD induction, respectively (Fig. 3a). CL administered at a dose of 1 mg/kg to IBD rats, resulted in a significant reduction in MaUS from 3.9 ± 0.4, (IBD, no CL) to 1.7 ± 0.6 at day 4. At day 7, a small but not significant increase was noted in the MaUS. CL, administered at a dose of 10 mg/kg, significantly reduced MaUS from 4.3 ± 0.3 (IBD, no CL) to 1.2 ± 0.2 at day 2 and from 3.9 ± 0.4 (IBD, no CL) to 1.3 ± 0.4 at day 4 of IBD. There was a non-significant reduction in MaUS at day 7 of IBD. CL, when administered at a dose of 100 mg/kg significantly reduced MaUS (1.7 ± 0.4 IBD) and 0.2 ± 0.17 (IBD) on day 2 and 7, respectively, when compared to IBD control at days 2 (4.3 ± 0.3) and 7 (1.3 ± 0.4). On day 4, CL at 100 mg/kg showed no significant effect on MaUS (Fig. 3b).

### Effect of curcuma longa (CL) on the histology and mean microscopic ulcer score (MiUS) in acetic acid induced-inflammatory bowel disease (IBD)

**Fig. 4:**
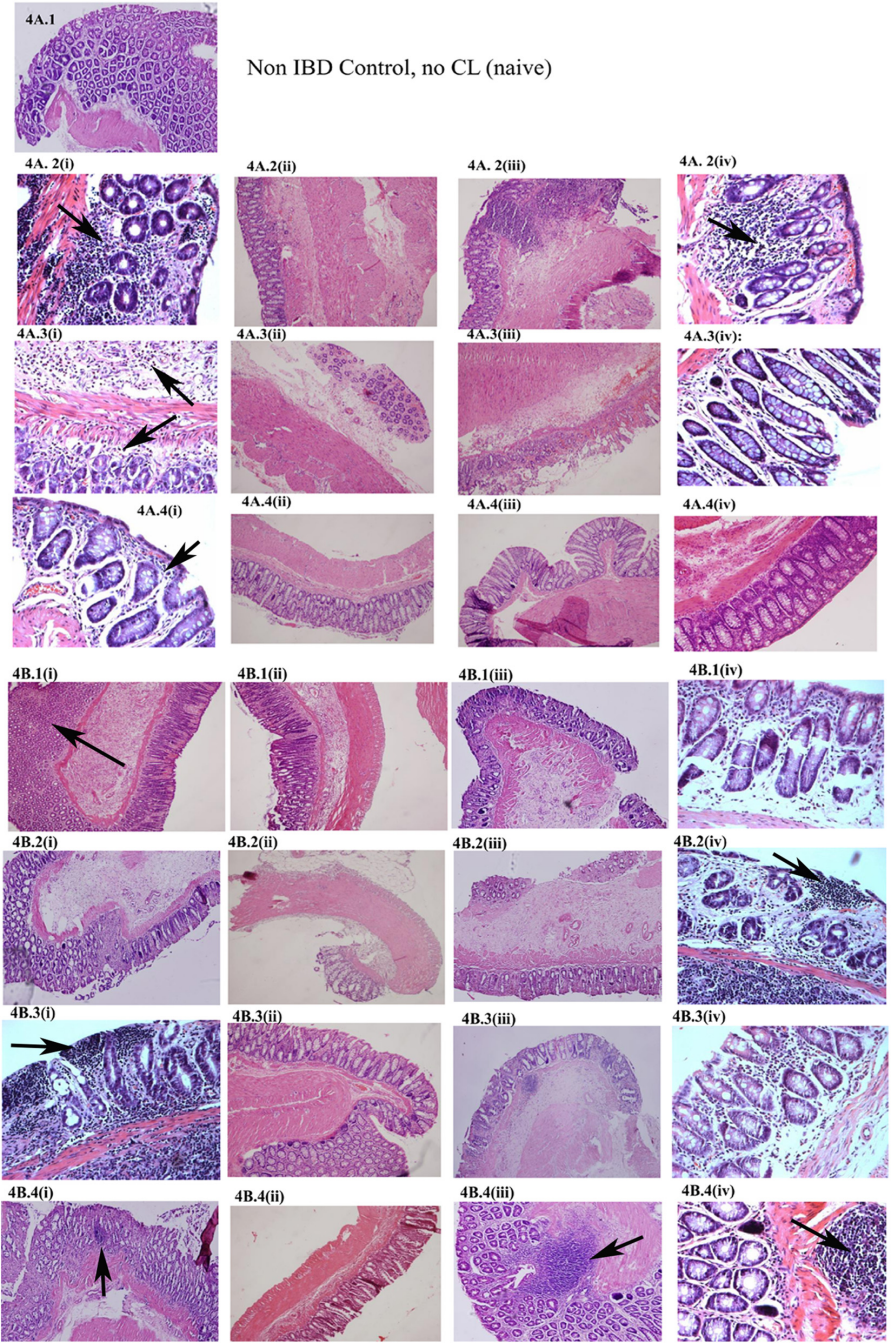
Figure 4A shows the effect of *Curcuma Longa* (CL) on the severity of inflammatory reactions in rats when given orally 30 min after the induction of IBD for a total of 7 days. The images show that severe lymphatic infiltration (arrow), especially those transcending all layers of the colon is more prevalent in the colon of untreated IBD rats compared to CL-treated animals. (*n =* 10). 4A. 1: Non IBD Control, no CL (naive). 4A. 2: 2 Days IBD, (i) Control, no CL, (ii) 1 mg/kg CL, (iii)10 mg/kg CL, (iv)100 mg/kg CL. 4A. 3: 4-day IBD (i) control, no CL, (ii)1 mg/kg CL, (iii) 10 mg/kg CL, (iv)100 mg/kg CL. 4A. 4: 7-day IBD (i) control, no CL, (ii) 1 mg/kg CL, (iii) 10 mg/kg CL, (iv) 100 mg/kg CL. Figure 4B shows the effect of CL on the microscopy of the colon of acetic acid-induced IBD rats 3 days before the induction of IBD. IBD caused a significant (*P <* 0.01) increase in the severity of leucocyte infiltration (arrow). CL administration at doses of 100 mg/kg did not markedly reduce the degree of leucocyte infiltration. 4B.1(i):Control, no CL, no IBD. 4B.1(ii): 1 mg/kg CL, no IBD, (iii): 10 mg/kg CL, no IBD, 4B.1(iv): 100 mg/kg CL, no IBD. 4B.2(i): no CL + 2 days IBD control; (ii): 1 mg/kg CL, (iii): 10 mg/kg CL + 2 days IBD, (iv): 100 mg/Kg + CL 2 days IBD. 4B.3(i): no CL + 4 days IBD control; (ii):1 mg/kg CL + 4 days IBD, (iii):10 mg/kg CL + 4 days IBD, (iv):100 mg/kgCL + 4 days IBD. 4B.4(i): no CL + 7 days IBD control; (ii): 1 mg/kg CL + 7 days IBD, (iii): 10 mg/kg CL + 7 days IBD, (iv):100 mg/kgCL + 7 days IBD

Administration of CL reduced lymphatic infiltration in the colon of rats with acetic acid induced-inflammatory bowel disease. This is more evident in the group treated with CL after the induction of IBD (Fig. 4a and b).

**Fig. 5:**
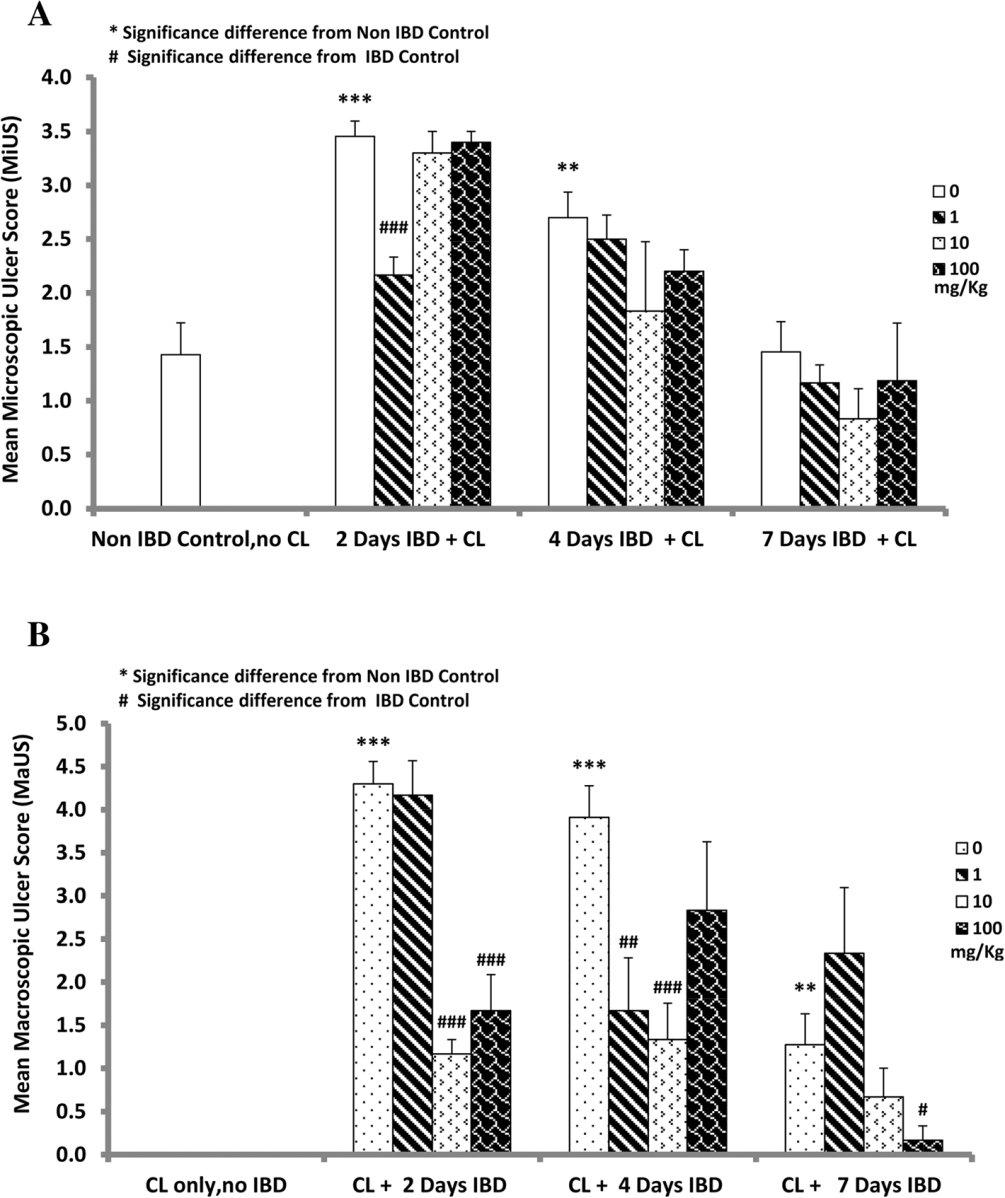
Figure 5 shows the effect of orally administered C*urcuma Longa* (CL) on microscopic ulcer score (MiUS) in rats before (**b**) and after (**a**) the induction of IBD. Note that CL, at doses of 1, 10 and 100 mg/kg caused a significant (*P <* 0.001) reduction in MiUS at different time points when compared to IBD control. *Significance difference between IBD and from Non IBD control; # Significance difference between CL-treated and IBD control (*n =* 6)

The results also show that MiUS was 1.4 ± 0.3 in the control group (No IBD, no CL). IBD caused a significant increase in MiUS when compared to saline control. The MiUS increased from 1.4 ± 0.3 to 3.5 ± 0.1 and 2.7 ± 0.2 at days 2 and 4, respectively. The effect tapered to almost normal at day 7. CL, at a dose of 1 mg/kg significantly reduced the MiUS (2.2 ± 0.2) when compared to control 3.5 ± 0.14 (IBD) at day 2. On days 4 and 7 of IBD, CL at 1 mg/kg had no significant effect on MiUS. CL, at doses of 10 and 100 mg/kg, non-significantly reduced MiUS on all days of IBD (Fig. 5).

The effect of CL on MiUS in rats when administered orally 3 days before the induction of IBD is shown in Fig. 5. IBD caused a significant (*P <* 0.01, *P <* 0.001) increase in MiUS to 3.5 ± 0.2 and 2.6 ± 0.3 on days 2 and 4, respectively compared to a baseline of 1.4 ± 0.3 (control, no CL, no IBD, *n =* 6). IBD did not cause any change to the MiUS on day 7. However, CL, at doses of 1, 10 and 100 mg/kg caused a significant reduction in MiUS on days 2 and 4 of IBD when compared to IBD control (no CL). Specifically, MiUS was 2.2 ± 0.2, 0.6 ± 0.08 and 1.5 ± 0.2 at doses of 1, 10 and 100 mg/kg, respectively on day 2 after the administration of CL compared to control, untreated IBD (3.5 ± 0.2). On day 4, MiUS was 0.8 ± 0.1 (*n =* 6), 0.9 ± 0.08 and 0.9 ± 0.8 after CL administration at doses of 1, 10 and 100 mg/kg, respectively when compared to untreated group (2.6 ± 0.3).

### Effect of curcuma longa (CL) on the mean serum glutathione (MSGSH) levels in acetic acid-induced inflammatory bowel disease (IBD)

**Fig. 6:**
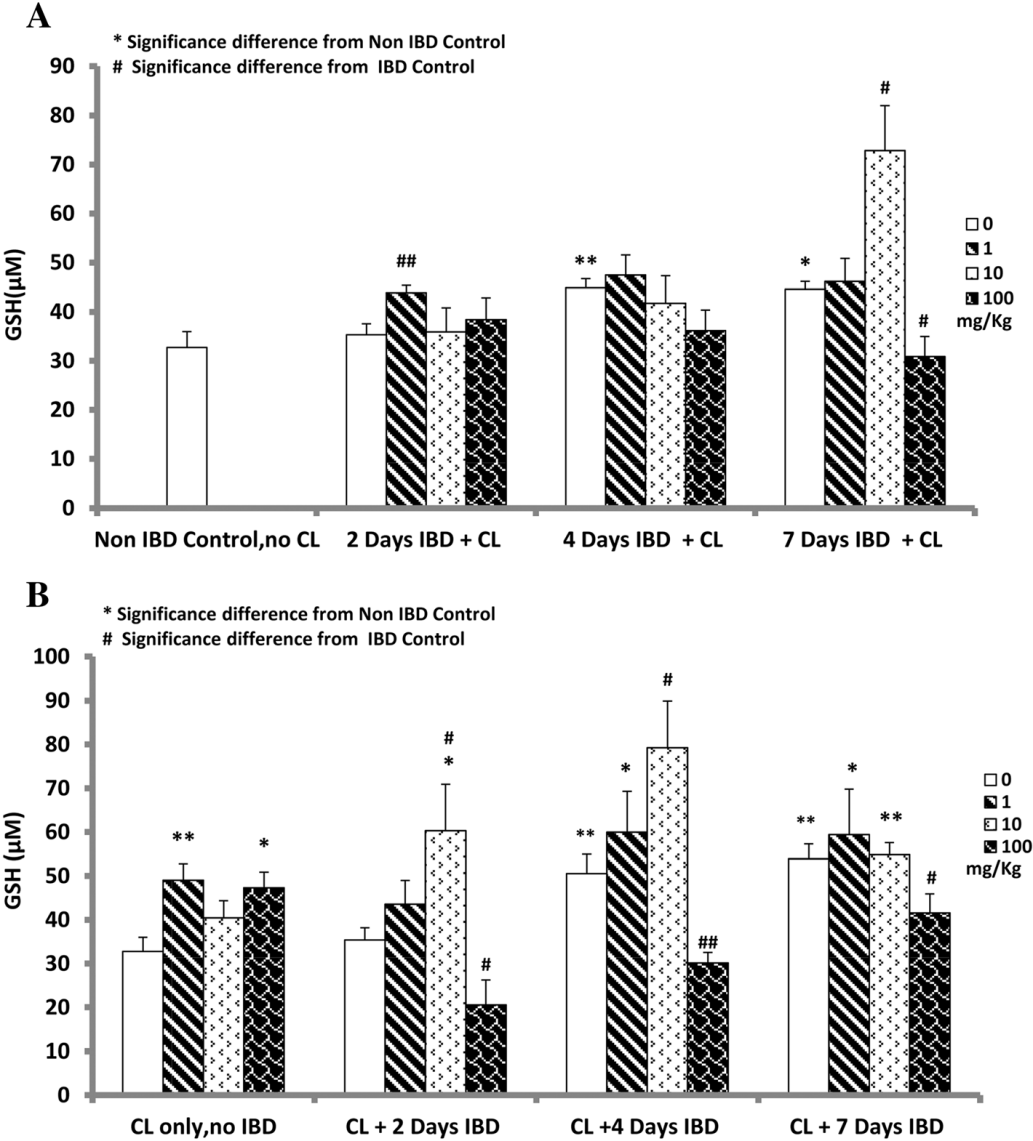
Figure 6 shows the effect of C*urcuma Longa* (CL) on mean serum glutathione (MSGSH) levels in rats before (**b**) and after (**a**) the induction of IBD. CL significantly (*P <* 0.05) increased MSGSH levels after 2 and 7 days of IBD compared to IBD control. *Significance difference between IBD and from Non IBD control; # Significance difference between CL-treated and IBD control (*n =* 6)

The effect of CL on MSGSH levels in rats when administered orally after the induction of IBD is shown in Fig. 6a. MSGSH in control (no IBD, no CL) was 33.0 ± 3.0 μM (*n =* 6). MSGSH levels increased significantly on days 4 and 7 of IBD. The MSGSH levels were 50.0 ± 1.8 μM and 45 ± 1.6 μM, respectively. CL significantly increased MSGSH levels after 2 (44 ± 1.5 μM, 1 mg/kg) and 7 (73 ± 9.0 μM, 10 mg/kg) days of IBD when compared to control. CL had no effect on MSGSH on day 4 of IBD at all doses tested. On day 7 of IBD, CL, at a dose of 100 mg/kg, significantly (*P <* 0.05) decreased MSGSH levels from 45 ± 1.6 μM (control IBD) to 31 ± 4.0 μM.

Figure 6b shows the effect of CL on MSGSH levels in rats, when given orally for 3 days before the induction of IBD. MSGSH in control (CL only, no IBD) was 33.0 ± 3.0 μM. IBD significantly increased MSGSH levels in control (no IBD, no CL) on days 4 and 7. The MSGSH levels increased from 33.0 ± 3.0 in control to 51 ± 4.0 μM (day 4) and 54 ± 3.0 μM (day 7). On day 2 of IBD, the value was not significantly different from that of positive control group (no IBD, CL only). CL significantly increased MSGSH levels on days 2 and 4 compared to untreated control (IBD, no CL). On day 2 of IBD, MSGSH increased from 35.0 ± 3.0 μM (IBD) to 60 ± 5.0 μM (CL 10 mg/kg). On day 4 of IBD, CL increased MSGSH levels from 54.0 ± 4.0 μM (Control IBD) to 79 ± 9.0 μM (CL 10 mg/kg). CL, administered at a dose of 1 mg/kg, had no effect on MSGSH levels compared to control (IBD, no CL). CL, administered at a dose of 100 mg/kg significantly reduced MSGSH levels on all days tested. It thus appear therefore that very low or high doses of CL decrease on the MSGSH level, while the average dose of 10 mg increase MSGSH significantly.

### Effect of curcuma longa (CL) on mean colon myeloperoxidase (MCMPO) levels in acetic acid-induced inflammatory bowel disease (IBD)

**Fig. 7:**
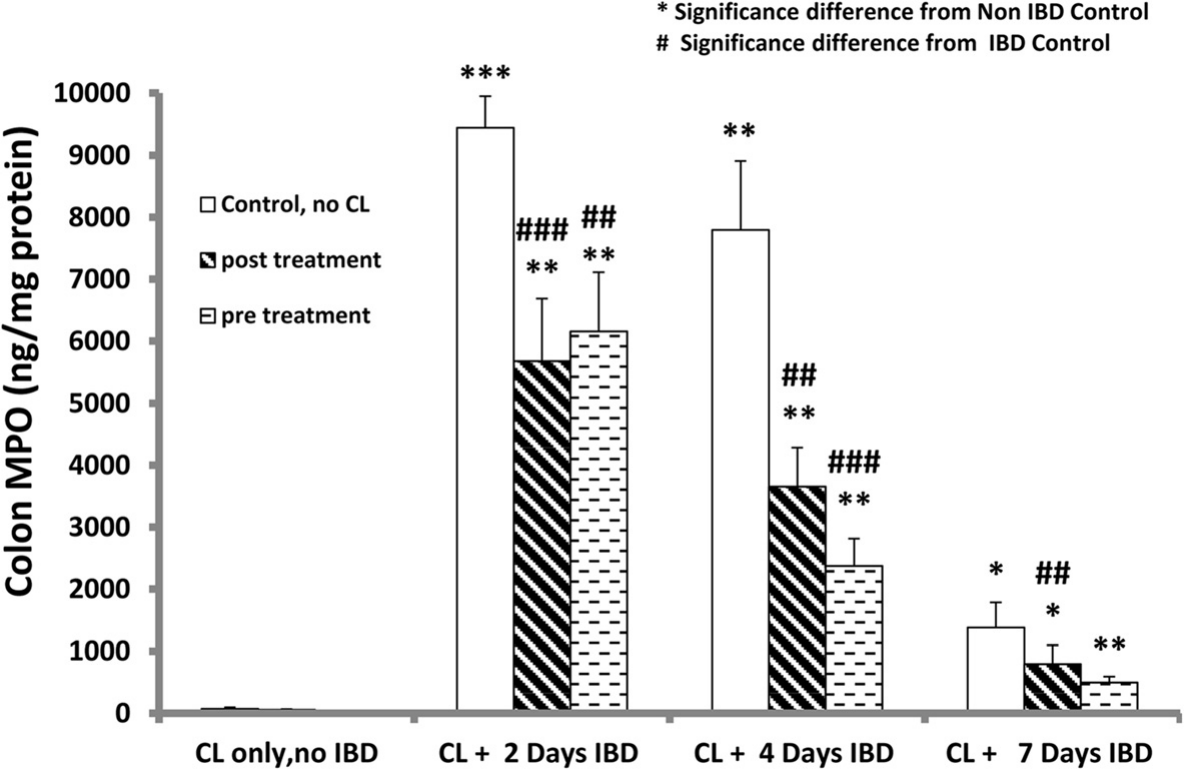
Figure 7 shows the effect of C*urcuma Longa* (CL) on mean colon myeloperoxidase (MPO) in IBD rat model levels in rats before and after the induction of IBD. CL significantly (*P <* 0.05) reduced MPO levels after 2, 4 and 7 days of IBD compared to IBD control. *Significance difference between IBD and from Non IBD control; # Significance difference between CL-treated and IBD control (*n =* 6)

The effect of CL on MCMPO levels in rats administered orally for 3 days before or 30 min after the induction of IBD was measured to determine the extent of inflammation (Fig. 7). MCMPO level in control (No CL, no IBD) was 74.0 ± 25.0 ng/mg protein. IBD significantly increased MCMPO levels to 9441 ± 510 ng/mg protein in untreated group (IBD, no CL) after 2 days of IBD. However, CL significantly reduced MCMPO levels from 9441 ± 510.0 (no CL) to 6157 ± 954 ng/mg of protein, from 7797 ± 1111 (no CL) to 2374 ± 442 ng/ml of protein and from 1381 ± 408 (no CL) to 501 ± 94 (*n =* 5) at 2, 4 and 7 days of IBD, respectively. When CL was post-administered 30 min after the induction of IBD, CL significantly reduced the MCMPO levels from 9441 ± 510 ng/ml of protein in untreated group (IBD, no CL) to 5680 ± 1008 ng/ml of protein, from 7797 ± 1111 to 3660 ± 626 ng/ml of protein and from 1381 ± 408 to 794 ± 302 ng/ml of protein at 2, 4 and 7 days of IBD, respectively.

### Effect of curcuma longa (CL) on mean colon IL 23 levels in acetic acid-induced inflammatory bowel disease (IBD)

**Fig. 8:**
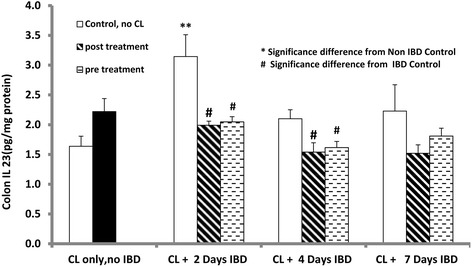
Figure 8 shows the effect of C*urcuma Longa* (CL) on mean colon IL-23 in IBD rat model levels in rats before and after the induction of IBD. CL significantly (*P <* 0.05) reduced IL-23 levels after 2 and 4 days of IBD compared to IBD control. *Significance difference between IBD and from Non IBD control; # Significance difference between CL-treated and IBD control (*n =* 6)

Colonic IL 23, a marker of inflammation, was measured in to examine the effect of CL after the induction of IBD (Fig. 8). Mean colonic IL 23 level before IBD in the control group (no IBD, no CL) was 1.6 ± 0.17 pg/mg of protein. IBD significantly increased mean colonic IL 23 levels to 3.14 ± 0.37 (IBD, no CL) pg/gm of protein. Pre-treatment with CL 3 days before IBD significantly reduced mean colonic IL 23 levels from 3.14 ± 0.37 (no CL) to 2.05 ± 0.09 pg/mg of protein, from 2.10 ± 0.15 to 1.62 ± 0.10 pg/mg of protein and non-significantly from 2.23 ± 0.44 to 1.81 ± 0.13 (*n =* 6) pg/mg of protein at 2, 4 and 7 days of IBD, respectively. Similarly when CL was administered 30 min after the induction of IBD, mean colonic IL 23 was significantly reduced from 3.14 ± 0.37 (no CL) to 1.99 ± 0.07 pg/mg of protein, from 2.10 ± 0.15 (no CL) to 1.54 ± 0.16 pg/mg of protein and non-significantly from 2.23 ± 0.44 (no CL) to 1.52 ± 0.14 pg/mg of protein at 2, 4 and 7 days of IBD, respectively.

## Discussion

Ulcerative colitis (UC) and Crohn’s disease (CD) are the two main types of inflammatory bowel disease (IBD). Patients can be become severely wasted during an acute attack of the disease [22]. Chronic undernourishment probably occurs more commonly in CD than in UC [23, 24]. The rat model of colonic inflammation was first developed by Morris et al., in 1989 [25] using intra-luminal instillation of trinitrobenzene sulfonic acid. In this study 1 ml of 4 % acetic acid was used, a known inducer of colonic inflammation. The results showed that IBD decreases body weight in rats, a similar trend seen in human studies. Several other studies have documented weight loss of up to 70–80 % of hospitalized patients [26]. According to Mijac et al. [27] weight loss may be an unreliable indicator, as it depends on the accuracy of previous weight measurement, patient’s memory, or water balance of patients. However, in this study, an animal model of IBD was used, which did not depend much on such complex parameters. Weight loss and malnutrition in IBD is multifactorial, and the nutritional status is the result of complex pathophysiological processes [28, 29]; these include postprandial pain, diarrhoea or anorexia, malabsorption and maldigestion due to either the active disease, protein loss through the bowel and metabolic stress associated with both inflammation and steroid therapy [24, 30, 31]. Diarrhoea was observed in all animals tested and 50 % of the IBD rats had blood in their stool, which is in line with the reports of other investigators [23, 29, 30]. Although IBD is generally treated with antiinflammatory or immunosuppressive drugs, most of these treatments are inadequate and consequently, many patients are turning to alternative treatments such as plant-based remedies. Food derivatives with plant-based remedies have an added advantage in that they are relatively nontoxic. Consequently, this study is focused on turmeric, the powdered rhizome of the medicinal plant, *Curcuma longa* Linn, used as a food flavouring and colouring agent in the Asian diet. It contains curcumin (diferuloylmethane), a polyphenolic pigment [16]. Curcumin has been shown to possess a variety of pharmacological effects including anti-inflammatory activities [17–20]. The results of the present study clearly indicate that turmeric has a protective effect on colitis-induced weight loss, a known parameter of colitis in humans and animals [21–23, 25–31]. This is the first report of its kind on the effect of turmeric on experimental colitis in rats using 4 % acetic acid. The only other similar study was on the antinociceptive effect of CL on acetic acid-induced writhing movement in mice [32], the effect of its active constituent, curcumin, on dinitrobenzene-induced experimental colitis [33] and the effect of curcumin, but not turmeric, on oxido-inflammatory regulation in a rat model of acetic acid-induced colitis [34]. Other studies on the effect of turmeric were done on experimental arthritis and not on experimental colitis. They observed that turmeric inhibited lipooxygenase and cyclooxygenase pathway and this could explain its inhibitory action in experimental colitis in this study [35–37]. Higher doses (100 mg/kg CL) caused a reduction in weight in this study which is similar to the observation of Deshpande et al. [38] who used a dose of 5 % CL for 90 days. Similar observation was made by Bille et al. [39] who used turmeric oleoresin for 102–109 days at doses of 60, 296 and 1551 mg/kg. The higher doses showed a reduction in weight gain.

In order to study the anti-inflammatory effect of CL, we used 4 % acetic acid, a known inducer of colitis [34, 40–42].

### Gross examination of acetic acid-induced colonic ulcer

Macroscopic damage was assessed by using the inflammation/ulcer scoring system of Wallace and Keenan [21]. This system takes into account the area of inflammation and the presence or absence of ulcers measured by macroscopic ulcer score (MaUS) which in turn is based on the semi quantitative scoring system. 6 cm of colon extending proximally from 2 cm above the anal margin was removed, split longitudinally, pinned out on a card, and the macroscopic appearances of the colonic mucosa scored as mentioned above. Most of the studies on CL have used curcumin (the active constituent, also referred to as curcumin 1 or diferuloylmethane) to test its anti-inflammatory effect, both *in vitro* and in *vivo* [43–45]. In a pilot, open-labeled, study conducted in New York, oral curcumin was given to 5 patients with ulcerative proctitis and 5 patients with Crohn’s disease (CD), all patients with proctitis and 80 % of patients with CD showed improvement [46]. Subsequently, other studies have shown similar anti-inflammatory potential of curcumin [47, 48]. Since CL also contains other active constituents namely desmethoxycurcumin (curcumin II), and bisdesmethoxycurcumin (curcumin III), as well as volatile oils, sugars, proteins, and resins [43], we decided to use the raw powdered rhizome of CL to study its effect in an animal model of inflammation. Our results show that CL reduced inflammation as measured by the MaUS. This is consistent with previous report [32, 49], which were done in adjuvant-induced arthritis (AIA), and carrageenan, dextran and formalin induced inflammation.

### Light microscopy

In order to get a better insight into the depth of inflammation, we undertook microscopic analysis of the ulcer, measured as microscopic ulcer score or MiUS. The microscopic analysis was based on the scoring system of Neurath et al. [5]. Randomly distributed full thickness biopsy specimens were used for this experiment. The results show that 4 % acetic acid caused a substantial degree of inflammation and tissue injury in the rat colon as demonstrated by the MiUS which in turn corresponds to an infiltration of the colon with leukocytes cells. Although macroscopic examination showed no evidence of any injury in control, microscopic observation showed some low level infiltration of the colon (no IBD, no CL). This is not unusual and commonly used bowel preparations can produce histological abnormalities of the colorectal mucosa [50, 51]. These might include surface erosions and leucocyte infiltration of the surface epithelium (aphthoid ulcers/lesions) and sometimes focal active cryptitis (FAC) as well as leucocyte infiltration of the subepithelial lamina propria [50, 52, 53]. The induction of IBD by 4 % acetic acid caused a substantial increase in transmural leukocytes infiltration, loss of goblet cells, high vascular density and thickening of the colonic wall. This is line with similar observation [34, 40, 41] done on acetic-acid- and 2,4,6-trinitrobenzene sulphonic acid–induced colitis in mice and rats. CL caused a significant reduction in the MiUS as demonstrated by reduced infiltration by leucocytes. This effect of CL in experimental acetic acid-colitis is unique in that others have shown anti-inflammatory and anti-oxidant effects of CL on carrageenan, dextran and formalin and adjuvant-induced arthritis, respectively [32, 49].

### Anti-oxidant effect of curcuma longa Linn

CL has been shown to have anti-oxidant and anti-inflammatory properties [32, 49] and curcumin, a yellow colouring agent extracted from CL, has also been shown to have anti-inflammatory and anti-oxidant activity [18–20]. There is a growing body of evidence implicating reactive oxygen metabolites as important mediators of inflammation-induced mucosal injury associated with IBD [49, 50]. Phagocytic leukocytes are most probably the source of these oxidants which are known to be present in large numbers in the inflamed mucosa and are known to produce large amounts of reactive oxygen species (ROS) in response to inflammation [54]. Because the gut mucosa contain relatively small amounts of antioxidant enzymes such as superoxide dismutase (SOD), catalase, glutathione (GSH) peroxidase, it is possible that it may be overwhelmed during times of active inflammation which could result in intestinal injury [54]. An imbalance between increased reactive oxygen species levels and decreased antioxidant defenses occurs in CD patients. Therefore, we undertook this study to determine whether serum levels of GSH is affected in acetic acid-induced colitis in rats. Our study showed that IBD increased serum GSH levels in control (no CL), which is in contrast to other who observed a reduction in GSH levels [41, 55, 56]. This is probably a compensatory mechanism to counter the inflammatory responses. Administration of CL significantly increased serum GSH levels which were higher and above the increase observed in control IBD (no CL) which is the first report of its kind in acetic acid-induced IBD. Other reports [49, 50] were on carrageen, dextran, formalin and adjuvant-induced arthritis.

### Inflammatory markers

Thus, CL acts as a protective mechanism counteracting the inflammation seen in our study.

Because of the presence of neutrophils in the mucosa and submucosa of the colon, we undertook to measure the colon myeloperoxidase (MPO) since it is extensively used as a biochemical marker for granulocyte (mainly neutrophil) infiltration into gastrointestinal tissues [25]. MPO is a glycosylated hemoprotein enzyme found in granules of human neutrophils and monocytes [57]. It is an important enzyme used during phagocytic lysis of engulfed foreign particles which takes part in the defense of the organism through production of hypochlorous acid (HOCl), a potent oxidant and an endogenous halide during phagocytosis, thereby contributing to the overall microbicidal and cytotoxic function of the polymorphonuclear leukocytes (PMN). MPO also modulates inflammation by generating ROS and thus contribute to the inflammation, which was evident in our study [58–60]. In this study, CL inhibited colonic MPO levels which indirectly reduces autooxidants and thus contribute to its overall anti-inflammatory properties.

The demonstration of the association of IL-23 pathway in multiple chronic inflammatory disorders, including inflammatory bowel disease (IBD) prompted us to measure the colonic IL 23 levels. Our results clearly showed that CL has a protective action against inflammation judging from its inhibitory actions on colonic IL 23. Evidence for the importance of IL-23 pathway in IBD has come from mouse models of IBD, in which IL-23 deficiency or blockade protects not only the mice from the disease [7, 8], but human IBD as well [9–11]. IL 23 does not stimulate interferon-γ (INF- γ) but acts on memory B and T cells and is thought to enhance CD4+ T cells that produce IL 17 [7].

### Treatment of C. longa before or after the onset of inflammatory bowel disease

The beneficial effects of C. *longa* on both macro- and macroscopic ulcer scoring, antioxidant pool, and level of inflammatory markers appear to be more conspicuous when administered before the onset of IBD. The differences may be due to the fact that a significant tissue damage may already have been done when *C. longa* is administered after the induction of IBD. The *C. longa* given may therefore not be able to repair the damage that has already been done to the colonic mucosa. In contrast, *C. longa*, when given before the onset of IBD is able to increase the tissue level of GSH, an endogenous antioxidant that is capable of reducing the level of oxidative stress generated by acetic acid. This is evident from the fact that the level of inflammatory markers is significantly higher after the administration of IBD. It thus appears that increased C. longa-induced GSH plays a part in the reduction of the deleterious effect of IBD and especially when it is given before the onset of the disease.

## Conclusion

In conclusion CL administration, either before or after the induction of IBD displays potent anti-inflammatory effects, as shown by the recovery in MBW, MaUS and MiUS and and inhibition of MPO and IL 23. All of these may suggest the possibility of developing *C. longa* as a safe and potent anti-inflammatory and antioxidant substance in the fight against IBD. 10 mg appears to be the ideal dose as very low (1 mg) or high (100 mg) do produce inconsistent outcomes. A possible reason for the inconsistencies with the very low or high doses is that the concentration of the active ingredients may be unevenly dispersed within the powder and leading to unequal amount of absorbed phytochemical.

### Limitations of the study

*C. longa* was observed to be beneficial at some doses while un-effective in others. The reason for this observation may be due to the fact that a powdered form of C. *longa* was used. A probable cause of this observation could be an uneven distribution of the active ingredients of C. *longa* powder. In addition, the absorption of C. *longa* after oral administration may not be even after all.

## References

[1] Kirsner JB, Shorter RG (1988). Inflammatory bowel disease.

[2] Sartor RB (1995). Current concepts of the etiology and pathogenesis of ulcerative colitis and Crohn’s disease. Gastroenterol Clin North Am.

[3] Van Deventer SJ (1997). Tumour necrosis factor and Crohn’s disease. Gut.

[4] Simpson SJ, Shah S, Comiskey M, de Jong YP, Wang B, Mizoguchi E, Bhan AK, Terhorst C (1998). T cell-mediated pathology in two model s of experimental colitis depends predominately on the interleukin 12/signal transducer and activator of transcription (STAT)-4 pathway, but is not conditional on interferon gamma expression by T cells. J Exp Med.

[5] Neurath MF, Fuss I, Kelsall BL, Stüber E, Strober W (1995). Antibodies to interleukin 12 abrogate established experimental colitis in mice. J Exp Med.

[6] Oppmann B, Lesley R, Blom B (2000). Novel p19 protein engages IL-12p40 to form a cytokine IL-23 with biological activities similar as well as distinct from IL-12. Immunity.

[7] Elson CO, Cong Y, Weaver CT (2007). Monoclonal anti-interleukin 23 reverses active colitis in a T cell-mediated model in mice. Gastroenterology.

[8] Kullberg MC, Jankovic D, Feng CG (2006). IL-23 plays a key role in *Helicobacter hepaticus-*induced T cell-dependent colitis. J Exp Med.

[9] Schmidt C, Giese T, Ludwig B (2005). Expression of interleukin-12-related cytokine transcripts in inflammatory bowel disease: elevated interleukin-23p19 and interleukin-23p28 in Crohn’s disease but not in ulcerative colitis. Inflamm Bowel Dis.

[10] Holtta V, Klemetti P, Siponnen T (2008). IL-23/Il-17 immunity as a hallmark of Crohn’s disease. Inflamm Bowel Dis.

[11] Saruta M, Yu QT, Fleshner PR (2007). Characterization of FOXP3 + CD4+ regulatory T cells in Crohn’s disease. Clin Immunol.

[12] Hanauer SB (1996). Inflammatory bowel disease. N Eng J Med.

[13] Sharma S, Strutzman JD, Kellof GJ, Steele VE (1994). Screening of potential chemotherapeutic agents using biochemical markers of carcinogenesis. Cancer Res.

[14] Ploto A (2003). Turmeric: post-production management for improved market access for herbs and spices-turmeric.

[15] Spices Board of India. Ministry of Commerce. Available online: http//www.indianspices.com/ (accessed on 20 February 2016).

[16] Cooper TH, Clarke G, Guzinski J. Teas, spices and herbs. In: Food Phytochemicals, ed. Ho, CT Vol. I pp 231–236, Washington, DC: American Chemical Society; 1994.

[17] Chuang SE, Cheng AL, Lin JK, Kuo ML (2000). Inhibition by curcumin of diethylnitrosamine-induced hepatic hyperplasia, inflammation, cellular gene products and cell-cycle-related proteins in rats. Food Chem Toxicol.

[18] Jian YT, Mai GF, Wang JD, Zhang YL, Luo RC, Fang YX (2005). Preventive and therapeutic effects of NF-kappa B inhibitor curcumin in rats colitis induced by trinitrobenzene sulfonic acid. World J Gastroenterol.

[19] Ukil A, Maity S, Karmakar S, Datta N, Vedasiromoni JR, Das PK (2003). Curcumin, the major component of food flavorturmeric, reduces mucosal injury in trinitrobenzene sulphonic acid induced colitis. Br J Pharmacol.

[20] Aldini R, Budriesi R, Roda G, Micucci M, Ioan P, D’Errico-Grigioni A, Sartini A, Guidetti E, Marocchi M, Cevenini M, Rosini F, Montagnani M, Chiarini A, Mazzella G (2012). Curcuma longa extract exerts a myorelaxant effect on the ileum and colon in a mouse experimental colitis model, independent of the anti-inflammatory effect. PLoS One.

[21] Wallace JL, Keenan CM (1990). An orally active inhibitor of leukotriene synthesis accelerates healing in a rat model of colitis. Am I Physiol.

[22] Silk DBA, Payne-James J (1989). Inflammatory bowel disease: nutritional implications and treatment. Proc Nutr Soc.

[23] Eiden KA (2003). Nutritional consideration in inflammatory bowel disease. Pract Gastroenterol.

[24] Gassull MA (2003). Nutritional consideration in inflammatory bowel disease: its relation to pathophysiology, outcome and therapy. Dig Dis.

[25] Morris GP, Beck PL, Herridge MS, Depew WT, Szewczuk MR, Wallace JL (1989). Hapten-induced model of chronic inflammation and ulceration in the rat colon. Gastroenterology.

[26] Lanfranchi GA, Brignola C, Campieri M, Bazzocchi G, Pasquali R, Bassein L, Labò G (1984). Assessment of nutritional status in Crohn’s disease in remission or low activity. Hepatogastroenterology.

[27] Mijac D, Janković GLJ, Jorga J, Krtić MN (2010). Nutritional status in patients with active inflammatory bowel disease: Prevalence of malnutrition and methods for nutritional assessment. Eur J Int Med.

[28] Jeejeebhoy KN (2002). Clinical nutrition: management of nutritional problems in of patients with Crohn’s disease. CMAJ.

[29] Goh J, O’Morain CA (2003). Nutrition and adult inflammatory bowel disease Review article. Alim Pharmacol Ther.

[30] Powell-Tuck J, Hennessy EM (2003). A comparison of mid upper arm circumference, body mass index and weight loss as indices of undernutrition in acutely hospitalized patients. Clin Nutr.

[31] Klein S, Meyers S, O’Sullivan P, Barton D, Leleiko N, Janovitz HD (1988). The metabolic impact of active ulcerative colitis. Energy expenditure and nitrogen balance. J Clin Gastroenterol.

[32] Ballinger AB, Azooz Q, El-Haj T, Poole S, Farthing MJ (2000). Growth failure occurs through a decrease in insulin-like-growth factor 1 which is independent of undernutrition in a rat model of colitis. Gut.

[33] Liju VB, Jeena K, Kuttan R (2011). An evaluation of antioxidant, anti-inflammatory, and antinociceptive activities of essential oil from Curcuma longa L. Indian J Pharmacol.

[34] Salh B, Assi K, Templeman V, Parhar K, Owen D (2003). Curcumin attenuates DNB-induced murine colitis. Am J Gastrointest Liver Physiol.

[35] Topcu-Tarladacalisir Y, Akpolat M, Uz YH, Kizilay G, Sapmaz-Metin M (2013). Cerkezkayabekir, Omurlu IK. Effects of curcumin on apoptosis and oxidoinflammatory regulation in a rat model of acetic acid-induced colitis: the roles of c-Jun N-terminal kinase and p38 mitogen-activated protein kinase. J Med Food.

[36] Funk JL, Frye JB, Oyarzo JN, Kuscuoglu N, Wilson J, McCaffrey G, Stafford G, Chen G, Lantz RC, Jolad SD, Sólyom AM, Kiela PR, Timmermann BN (2006). Efficacy and mechanism of action of turmeric supplements in the treatment of experimental arthritis. Arthritis Rheum.

[37] Funk JL, Oyarzo JN, Frye JB, Chen G, Lantz RC, Jolad SD, Sólyom AM, Timmermann BN (2006). Turmeric extracts containing curcominoids prevent experimental rheumatoid arthritis. J Nat Med.

[38] Rao CV (2006). Turmeric natural COX 2 inhibitor. Adv Exp Med Biol.

[39] Deshpande SS, Lalitha VS, Ingle AD, Raste AS, Gadre SG, Maru GB (1998). Subchronic oral toxicity of turmeric and ethanolic turmeric extract in female mice and rats. Toxicol Lett.

[40] Bille N, Larsen JC, Hansen EV, Würtzen G (1985). Subchronic oral toxicity of turmeric oleoresin in pigs. Food Chem Toxicol.

[41] Yamada T, Zimmerman BJ, Specian RD, Grisham MB (1991). Role of neutrophils in acetic acid-induced colitis in rats. Inflammation.

[42] Millar AD, Rampton DS, Chander CL, Claxson AWD, Blades S, Coumbe A, Panetta J, Morris CJ, Blake DR (1996). Evaluating the antioxidant potential of new treatments for inflammatory bowel disease using a rat model of colitis. Gut.

[43] El-Abhar HS, Hammad LN, Gawad HS (2008). Modulating effect of ginger extract on rats with ulcerative colitis. J Ethnopharmacol.

[44] Singh G, Kapoor IP, Singh P, de Heluani CS, de Lampasona MP, Catalan CA (2010). Comparative study and chemical composition and antioxidant activity of fresh and dry rhizomes of turmeric (Curcuma longa Linn.). Food Chem Toxicol.

[45] Camacho-Barquero L, Villegas I, Sάnchez-Calvo JM, Talero E, Sanchez-Fidalgo S, Motilva V (2007). Alarcón de la Lastra C. Curcumin. A Curcuma longa constituent, acts on MAPK p38 pathway modulating COX-2 and iNOS expression in chronic experimental colitis. Int Immunopharmacol.

[46] Sugimoto K, Hanai H, Tozawa K, Aoshi T, Uchijima M, Nagata T, Koide Y (2002). Curcumin prevents and ameliorates trinitrobenzenesulfonic acid-induced colitis in mice. Gastroenterology.

[47] Holt PR, Katz S, Kirshoff R (2005). Curcumin therapy in inflammatory bowel disease: a pilot study. Dig Dis Sci.

[48] Satoskar RR, Shah SJ, Shenoy SG (1986). Evaluation of anti-inflammatory property of curcumin (diferuloylmethane) in patients with postoperative inflammation. Int J Clin Pharmacol Ther Toxicol.

[49] Hanai H, Iida T, Takeuchi K, Watanabe F, Maruyama Y, Andoh A, Tsujikawa T, Fujiyama Y, Mitsuyama K, Sata M, Yamada M, Iwaoka Y, Kanke K, Hiraishi H, Hirayama K, Arai H, Yoshii S, Uchijima M, Nagata T, Koide Y (2006). Curcumin maintenance therapy for ulcerative colitis: randomized, multicenter, double-blind, placebo-controlled trial. Clin Gastroenterol Hepatol.

[50] Ramadan G (2011). Al–Kahtani MA, El-Sayed. Anti-inflammatory and anti-oxidant properties of Curcuma longa (turmeric versus Zingiber officinale (ginger) rhizomes in rat adjuvant-induced arthritis. Inflammation.

[51] Duncan JE, Quietmeyer CM (2009). Bowel preparation: current status. Clin Colon Rectal Surg.

[52] Pockros PJ, Foroozan P (1985). Golytely lavage versus a standard colonoscopy preparations. Effect on normal colonic mucosal histology. Gastroenterology.

[53] Greenson JK, Stern RA, Carpenter SL, Barnett JL (1997). The clinical significance of focal active colitis. Hum Pathol.

[54] Zwas FR, Nicholas W, Cirillo DE, Eisen RN (1996). Colonic mucosal abnormalities with oral sodium phosphate solution. Gastrointest Endosc.

[55] Kruidenier L, Verspaget HW (2002). Oxidative stress as a pathogenic factor in inflammatory bowel disease — radicals or ridiculous?. Alim Pharmacol Ther.

[56] AlZoghaibi MA (2013). Concepts of oxidative stress and anti-oxidant defense in Crohn’s disease. World J Gastroenterol.

[57] Yamada T, Grisham MB (1991). Role of neutrophil–derived oxidants in the pathogenesis of intestinal inflammation. Klin Wochenschr.

[58] Sido B, Hack V, Hochlehner A, Lipps H, Herfarth C, Droge W (1998). Impairment of intestinal glutathione synthesis in patients with inflammatory bowel disease. Gut.

[59] Tobler A, Koeffler HP, Harris JR (1991). Myeloperoxidase: localization, structure and function. Blood Cell Biochemistry.

[60] Clarke RA, Borregaard N (1985). Neutrophils autoinactivate secretory products by myeloperoxidase-catalyzed oxidation. Blood.

